# Examining national and district-level trends in neonatal health in Peru through an equity lens: a success story driven by political will and societal advocacy

**DOI:** 10.1186/s12889-016-3405-2

**Published:** 2016-09-12

**Authors:** Luis Huicho, Carlos A. Huayanay-Espinoza, Eder Herrera-Perez, Jessica Niño de Guzman, Maria Rivera-Ch, Maria Clara Restrepo-Méndez, Aluisio J. D. Barros

**Affiliations:** 1 Centro de Investigación para el Desarrollo Integral y Sostenible (CIDIS), Universidad Peruana Cayetano Heredia, Lima, Peru; 2 School of Medicine, Universidad Peruana Cayetano Heredia and Universidad Nacional Mayor de San Marcos, Lima, Peru; 3 Instituto Nacional de Salud del Niño, Lima, Peru; 4 Ministerio de Economía y Finanzas, Lima, Peru; 5 International Center for Equity in Health, Federal University of Pelotas, Pelotas, Brazil

**Keywords:** Neonatal mortality, Success factors, Policy and system analysis, Advocacy, Equity, Evidence-based interventions

## Abstract

**Background:**

Peru has impressively reduced its neonatal mortality rate (NMR). We aimed, for the period 2000–2013, to: (a) describe national and district NMR variations over time; (b) assess NMR trends by wealth quintile and place of residence; (c) describe evolution of mortality causes; (d) assess completeness of registered mortality; (e) assess coverage and equity of NMR-related interventions; and (f) explore underlying driving factors.

**Methods:**

We compared national NMR time trends from different sources. To describe NMR trends by wealth quintiles, place of residence and districts, we pooled data on births and deaths by calendar year for neonates born to women interviewed in multiple surveys. We disaggregated coverage of NMR-related interventions by wealth quintiles and place of residence. To identify success factors, we ran regression analyses and combined desk reviews with qualitative interviews and group discussions.

**Results:**

NMR fell by 51 % from 2000 to 2013, second only to Brazil in Latin America. Reduction was higher in rural and poorest segments (52 and 58 %). District NMR change varied by source. Regarding cause-specific NMRs, prematurity decreased from 7.0 to 3.2 per 1,000 live births, intra-partum related events from 2.9 to 1.2, congenital abnormalities from 2.4 to 1.8, sepsis from 1.9 to 0.8, pneumonia from 0.9 to 0.4, and other conditions from 1.2 to 0.7. Under-registration of neonatal deaths decreased recently, more in districts with higher development index and lower rural population. Coverage of family planning, antenatal care and skilled birth attendance increased more in rural areas and in the poorest quintile. Regressions did not show consistent associations between mortality and predictors. During the study period social determinants improved substantially, and dramatic out-of-health-sector and health-sector changes occurred. Rural areas and the poorest quintile experienced greater NMR reduction. This progress was driven, within a context of economic growth and poverty reduction, by a combination of strong societal advocacy and political will, which translated into pro-poor implementation of evidence-based interventions with a rights-based approach.

**Conclusions:**

Although progress in Peru for reducing NMR has been remarkable, future challenges include closing remaining gaps for urban and rural populations and improving newborn health with qualified staff and intermediate- and intensive-level health facilities.

**Electronic supplementary material:**

The online version of this article (doi:10.1186/s12889-016-3405-2) contains supplementary material, which is available to authorized users.

## Background

Peru met the fourth Millennium Development Goal (MDG) of reduction of under-five mortality rate by two thirds between 1990 and 2015 [1, 2], and is on track to meet the fifth goal of reduction of maternal mortality by three quarters by 2015 [1, 2]. It has also reduced dramatically under-five stunting and underweight prevalence, and has been ranked as the first country among all low and middle-income countries (LMIC) in reducing its neonatal mortality rate from 1990 onwards [3].

Initial global emphasis on child survival has recently shifted to focus on maternal and neonatal health, as an imperative to reach sustainable progress at country and local level [4–6]. Systematic analyses on factors driving progress in LMIC are critical to guide future policies and programmes. A recent multi-country review of health-systems bottlenecks and success factors related to country progress of neonatal mortality has identified health workforce planning, financial protection measures and dynamic leadership as key facilitating strategies for reduction of neonatal mortality in eight of the 13 countries accounting for the highest burden of neonatal deaths [7].

In-depth analysis disaggregated by wealth quintiles, urban and rural residences and by districts (equivalent to departments in Peru) may highlight a differing pace of progress and may help to identify key areas for further policy and programmatic refinement to ensure continued progress in newborn health.

We aimed to analyse national and district level neonatal mortality rate (NMR) from 2000 to 2013. We specifically aimed to: (a) describe national and district NMR variations over time; (b) assess NMR trends by wealth quintile and place of residence; (c) describe evolution of mortality causes; (d) assess completeness of registered mortality; (e) assess coverage and equity of NMR-related interventions; and (f) explore underlying driving factors.

## Methods

To describe national time trends, we compared neonatal deaths and rates from different sources including the United Nations Inter-agency Group for Child Mortality Estimation (IGME) [8], the Institute of Health Metrics and Evaluation (IHME) [9], the Child Health Epidemiology Reference Group [10, 11], those estimated through pooled data on births and deaths by calendar year for newborns born to women interviewed in multiple Demographic and Health Surveys (DHS) [12], and official registered & corrected neonatal mortality data [13, 14]. The study period was 2000–2013 for data from IGME, IHME and for individual DHS and, 2001–2011 for official registered and corrected rates. For national level birth cohorts-based NMR, baseline and last period were 1999–2001 and 2011–2013, respectively.

For NMR equity analyses by wealth quintiles and by urban and rural domains [15], NMRs based on birth cohorts were calculated from multiple DHS for five three-year periods, from 1999–2001 to 2011–2013 [16].

For district-level analyses, NMRs were also calculated through the pooled birth cohorts’ method, but in this case for four three-year periods, from 2001–2003 to 2010–2012, to accumulate sufficient data available at that level.

Average annual rates for coverage and equity of interventions related to NMR (e.g., family planning needs satisfied, at least 4 antenatal care visits, and skilled birth attendance) and for NMR by wealth quintiles and by urban and rural residences were determined through variance-weighted regression of log-rates on year, using the whole time series from the Peruvian DHS [15]. We compared average annual rates between geographic areas and between poorest and richest quintiles by testing for interaction between year and region.

To assess the completeness of registered and corrected neonatal deaths from the official registration system, we used the Child Health Epidemiology Reference Group (CHERG) information as the reference standard at national level [11], and the most recent registered and corrected neonatal deaths available for the 2011–2012 period as the reference at district-level [17]. We correlated the district under-registration degree with the proportion of urban population estimated by the Peruvian DHS [16], and with the human development index (HDI) [9] reported by the United Nations [18].

Data related to expenditure on reproductive, maternal and neonatal interventions were derived from the Ministry of Finance official website [19]. We used currency exchange rates from the World Development Indicators database and the population estimated by the Peruvian National Institute of Statistics to calculate per capita expenditure in constant 2012 US$ [20, 21].

To account for possible driving factors that may explain the neonatal mortality reduction over the study period, we estimated crude and adjusted coefficients through simple and multiple regressions, where the dependent variable was NMR and the covariates were individual factors from the different dimensions of an explanatory conceptual framework that we designed in advance. The conceptual framework encompassed four dimensions of factors that could explain the progress achieved in neonatal mortality, namely: contextual factors (GDP per capita in USD 2012 US$, Gini for income index, percentage of families living under the poverty line, percentage of families with at least one unmet basic need, percentage or urban population, median years of women’s schooling) obtained from the annual national household surveys and from the Peruvian DHS [12, 16], non-health sector changes (total fertility rate, percentage of households with piped water inside the house, rural coverage of JUNTOS conditional cash transfer programme) obtained from the DHS and from government reports [16, 22], health-sector changes (utilisation of the Comprehensive Health Insurance System SIS-number of annual under-five outpatient preventative and clinical attendances per total under-five population, density of doctors, nurses and midwives per 10,000 population) also obtained from government reports (http://www.sis.gob.pe/portal/index.html, [23]), and coverage of NMR-related interventions (percentage of women aged 15–49, either married or in union, who have their needs for family planning satisfied; percentage of pregnant women with at least four antenatal care visits; percentage of live births attended by skilled birth attendant) obtained from the Peruvian DHS [16].

We additionally included a qualitative analysis that encompassed several rounds of individual interviews and group discussions with people familiar with maternal and newborn health, complemented by a desk review. We targeted members of the academia, central and regional governments, non-governmental organisations, civil society representatives, and other stakeholders. The discussions focused on the potential underlying factors that could explain the progress in NMR reduction achieved by Peru, widely categorised in social determinants of health, non-health sector changes, health sector changes including RMNCH financing, and coverage of NMR-related interventions.

We sought to build a diagrammatic summary of the health policy and system changes that occurred in Peru, based on the information gathered, to support our analysis of contextual, policy, programmes and financing factors influencing the achieved NMR reduction.

## Results

### National time trends of neonatal mortality

**Fig. 1:**
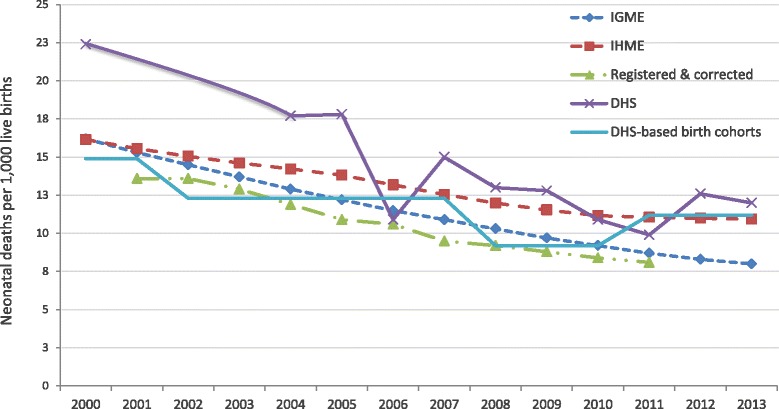
Neonatal mortality rate from 2000 to 2013 in Peru. Sources: IGME (UN Inter-agency Group for Child Mortality Estimation); IHME (Institute for Health Metrics and Evaluation); and DHS (Peruvian Demographic and Health Survey)

**Fig. 2:**
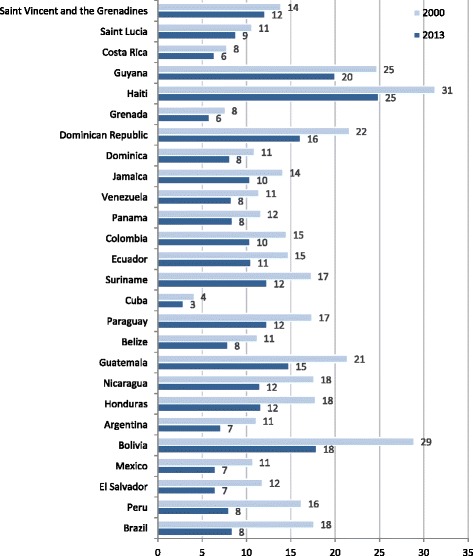
Neonatal mortality rate per 1000 live births in Latin American and Caribbean countries, 2000 and 2013. Source: IGME (Inter-agency Group for Child Mortality Estimation)

Figure 1 shows national NMR trends by five different sources. The percentage of reduction was 51 % for IGME-based NMR [8], 32.2 % for IHME [9], and 46.4 % for individual DHS [16]. Reduction percentages were 40.4 % for registered NMR [13, 14], and 24.8 % for NMR based on multiple DHS birth cohorts [16]. As for the percentage of reduction within the Latin American region from 2000 to 2013, Peru ranked only second to Brazil, according to IGME data (Fig. 2) [8].

**Fig. 3:**
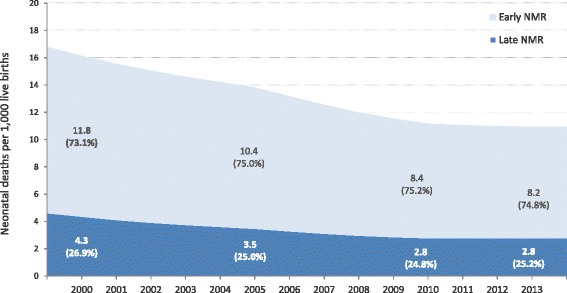
Trends in early and late neonatal mortality rate in Peru (2000–2013). Source: IHME (Institute of Health Metrics and Evaluation)

Figure 3 shows the reduction of early and late NMR over time according to IHME [9]. Early NMR accounted for 73.1 % of total NMR in 2000 and 74.8 % in 2013.

### National neonatal mortality profiles by cause

**Fig. 4:**
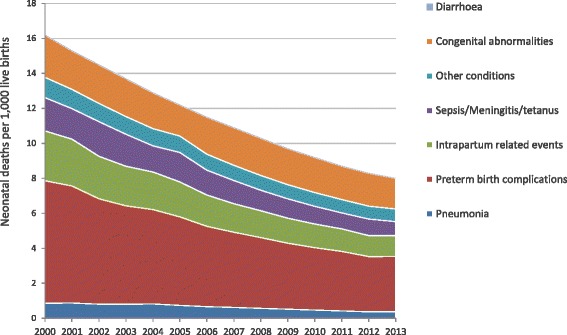
Neonatal mortality rates by specific cause in Peru (2000–2013). Sources: CHERG for deaths by cause, and IGME for neonatal mortality rates. CHERG, Child Health Epidemiology Reference Group

Figure 4 shows the reduction of cause specific NMR from 2000 to 2013 [8, 11]. For the year 2000, the figures were 16.2 per 1,000 live births for all causes; 7.0 for pre-term birth complications; 2.8 for intra-partum related events; 2.4 for congenital abnormalities; 1.9 for sepsis/meningitis and tetanus; 0.9 for pneumonia, and 1.2 for other conditions. As for the year 2013, NMR for all causes was 8.0; 3.2 for pre-term birth complications; 1.2 for intra-partum related events; 1.8 for congenital abnormalities; 0.8 for sepsis/meningitis and tetanus; 0.4 for pneumonia; and 0.7 for other conditions. Detailed annual NMR data by cause are provided by year as Additional file 1: Table S1.

### District-level time trends of neonatal mortality

District time trends of NMR (DHS-based birth cohorts) show that 19 out of 24 districts reduced their NMR, except for 5 (Arequipa, San Martin, La Libertad, Lima, Ayacucho), which actually increased them (Table 1). Districts that decreased more their NMR included Tacna, Pasco, Moquegua, Apurimac, and Ucayali (Table 1). No clear differences in patterns of NMR variation over time were found between departments when categorised by geographic region (Coast, Andes, Amazon) (Table 1). Among the districts that increased their NMR, Lima had a 22 % increase from 5.9 in 2001–2003 to 7.2 from 2010 to 2012 although registering a very low rate of 2.8 in the previous period, while Arequipa showed a 128 % increase from 2.8 to 6.3, and San Martin a 41.4 % increase from 16.8 to 23.7 during the same periods. Among the fast progressing districts, Tacna had a 100 % reduction of NMR during the same periods described above, followed by Pasco with 83 %, Moquegua with 64.6 %, Apurimac with 62.5 %, and Ucayali with 59.0 % of reduction (Table 1).

**Table 1:** Variations in district-level neonatal mortality over time by Demographic and Health Survey-based birth cohorts in Peru

Department	Region^a^	2001–2003	2004–2006	2007–2009	2010-2012	% of reduction
Amazonas	Amazon	14.26	13.34	11.05	13.99	1.8
Ancash	Andes	13.91	12.02	7.07	6.65	52.2
Apurimac	Andes	18.85	15.45	6.05	7.06	62.5
Arequipa	Andes	2.76	12.71	9.83	6.28	−128.0
Ayacucho	Andes	15.65	10.91	5.08	17.33	−10.8
Cajamarca	Andes	17.23	12.68	13.26	12.56	27.1
Cusco	Andes	15.12	15.49	9.09	14.73	2.6
Huancavelica	Andes	18.25	16.20	9.54	13.67	25.1
Huanuco	Andes	16.30	12.43	11.54	9.19	43.6
Ica	Coast	8.84	10.99	15.47	6.64	24.9
Junin	Andes	19.62	12.25	7.19	8.76	55.3
La Libertad	Coast	7.50	12.17	8.22	10.28	−37.0
Lambayeque	Coast	11.21	8.12	13.80	6.14	45.3
Lima/Callao	Coast	5.93	10.50	2.80	7.24	−22.0
Loreto	Amazon	24.28	19.25	13.68	18.70	23.0
Madre de Dios	Amazon	14.87	10.64	19.79	10.35	30.4
Moquegua	Coast	17.01	11.55	8.68	6.02	64.6
Pasco	Andes	18.20	21.48	12.92	3.03	83.4
Piura	Coast	14.61	13.80	12.59	7.09	51.5
Puno	Andes	21.32	15.00	16.65	12.36	42.0
San Martin	Amazon	16.78	15.57	14.54	23.74	−41.4
Tacna	Coast	12.53	9.51	8.81	0.00	100.0
Tumbes	Coast	13.09	20.81	12.68	12.55	4.1
Ucayali	Amazon	12.71	15.91	9.69	5.21	59.0

^a^Source: National Institute of Statistics and Computing (http://www.inei.gob.pe/media/MenuRecursivo/publicaciones_digitales/Est/Lib0015/cap-51.htm)

Table 2 shows rankings of districts performance by their annual or period progress of NMR, which differed widely depending on the source of mortality. For instance, for NMR variation by period, estimated from DHS-based birth cohorts, the districts with the highest annual reduction were Pasco, Apurimac, Tacna, Ucayali, and Moquegua (range of annual NMR reduction from 0.93 to 1.76); while three districts actually increased their annual NMR (San Martin, Ayacucho, and La Libertad) (range of annual NMR increase from 0.02 to 0.70).

**Table 2:** Ranking of districts in Peru by annual reduction of neonatal mortality rates

Department	Demographic and Health Survey (DHS)-based birth cohorts			Registered and corrected			Individual DHS		
	Ranking	Beta	SE	Ranking	Beta	SE	Ranking	Beta	SE
Amazonas	21	−0.04	0.13	1	−1.57	0.74	13	−0.73	0.36
Ancash	7	−0.74	0.13	9	−0.91	0.19	7	−1.19	0.56
Apurimac	2	−1.21	0.26	2	−1.55	0.13	4	−1.90	0.85
Arequipa	20	−0.07	0.33	12	−0.70	0.15	10	−0.89	0.49
Ayacucho	23	0.21	0.50	7	−1.03	0.20	6	−1.34	0.38
Cajamarca	14	−0.28	0.10	19	−0.25	0.14	11	−0.80	0.60
Cusco	18	−0.18	0.28	6	−1.16	0.20	1	−2.44	0.77
Huancavelica	11	−0.51	0.25	3	−1.44	0.35	2	−2.44	0.68
Huanuco	10	−0.57	0.06	4	−1.41	0.28	5	−1.87	0.58
Ica	16	−0.20	0.35	17	−0.31	0.24	12	−0.77	0.96
Junin	6	−0.89	0.21	8	−0.92	0.20	8	−1.18	0.93
La Libertad	22	0.02	0.18	21	0.33	0.12	3	−1.91	0.83
Lambayeque	15	−0.27	0.31	18	−0.28	0.09	14	−0.67	0.56
Lima/Callao	17	−0.19	0.30	14	−0.49	0.08	19	−0.48	0.28
Loreto	13	−0.48	0.28	22	0.55	0.60	22	−0.37	0.74
Madre de Dios	19	−0.10	0.43	20	0.24	0.34	23	−0.12	0.62
Moquegua	5	−0.93	0.08	10	−0.85	0.29	18	−0.51	0.37
Pasco	1	−1.76	0.43	13	−0.56	0.37	20	−0.43	0.70
Piura	8	−0.72	0.17	16	−0.37	0.14	21	−0.37	0.38
Puno	9	−0.61	0.16	5	−1.30	0.35	17	−0.53	1.01
San Martin	24	0.70	0.32	23	0.57	0.57	9	−1.08	0.48
Tacna	3	−1.12	0.25	11	−0.79	0.30	24	0.02	0.32
Tumbes	12	−0.49	0.34	15	−0.40	0.23	16	−0.57	0.60
Ucayali	4	−0.97	0.27	24	1.11	0.40	15	−0.67	0.51

**Fig. 5:**
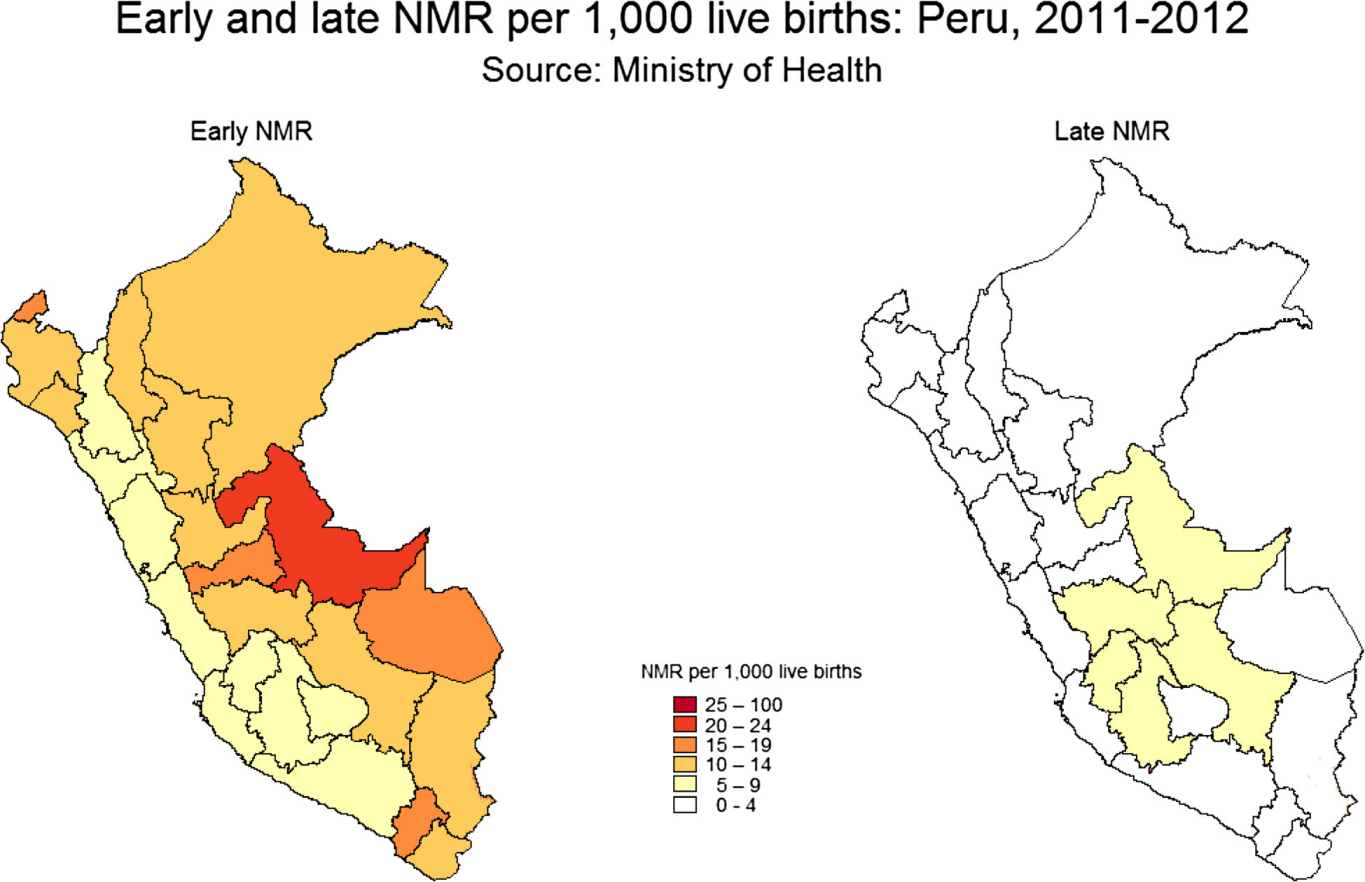
Early and late neonatal mortality rates in Peru (2011–2012) [16]

For early and late neonatal mortality rates at district-level, registered and corrected data for the period 2011–2012 revealed that most deaths occurred during the first seven days of life (Fig. 5). Fifteen districts showed early NMR of 10 per 1,000 live births and more (Fig. 5) [17]. Detailed information is provided in Additional file 1: Table S2.

### Equity analyses of mortality and coverage

**Fig. 6:**
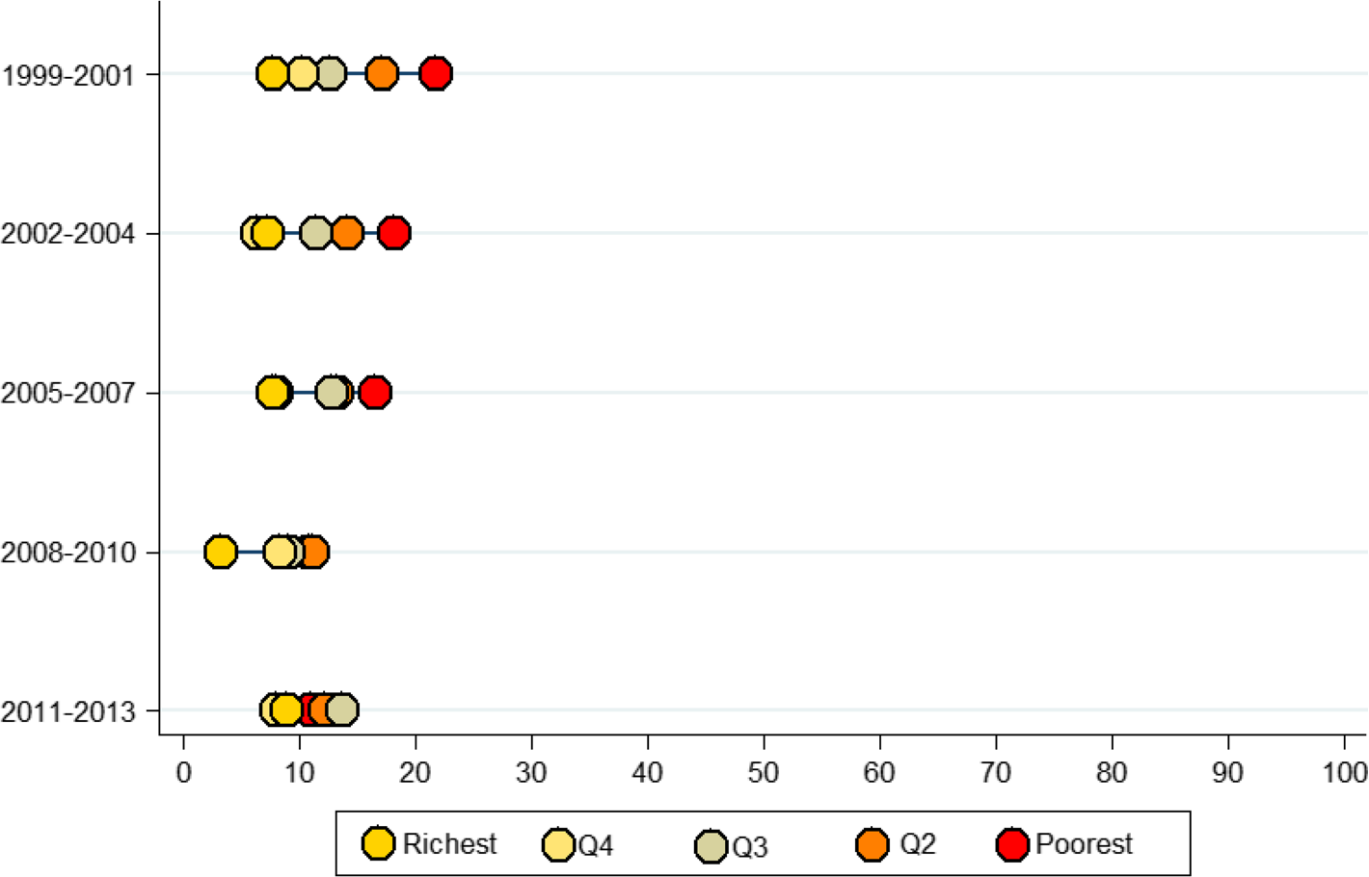
Neonatal mortality rate (Demographic and Health Survey-based birth cohorts) by wealth quintiles in Peru

For the period 1999–2001, the NMR for the richest and poorest quintiles were 7.8 and 21.9 per 1,000 live births, respectively, while for the period 2011–2013 they were 9.1 and 11 per 1,000 live births, respectively (Fig. 6).

**Fig. 7:**
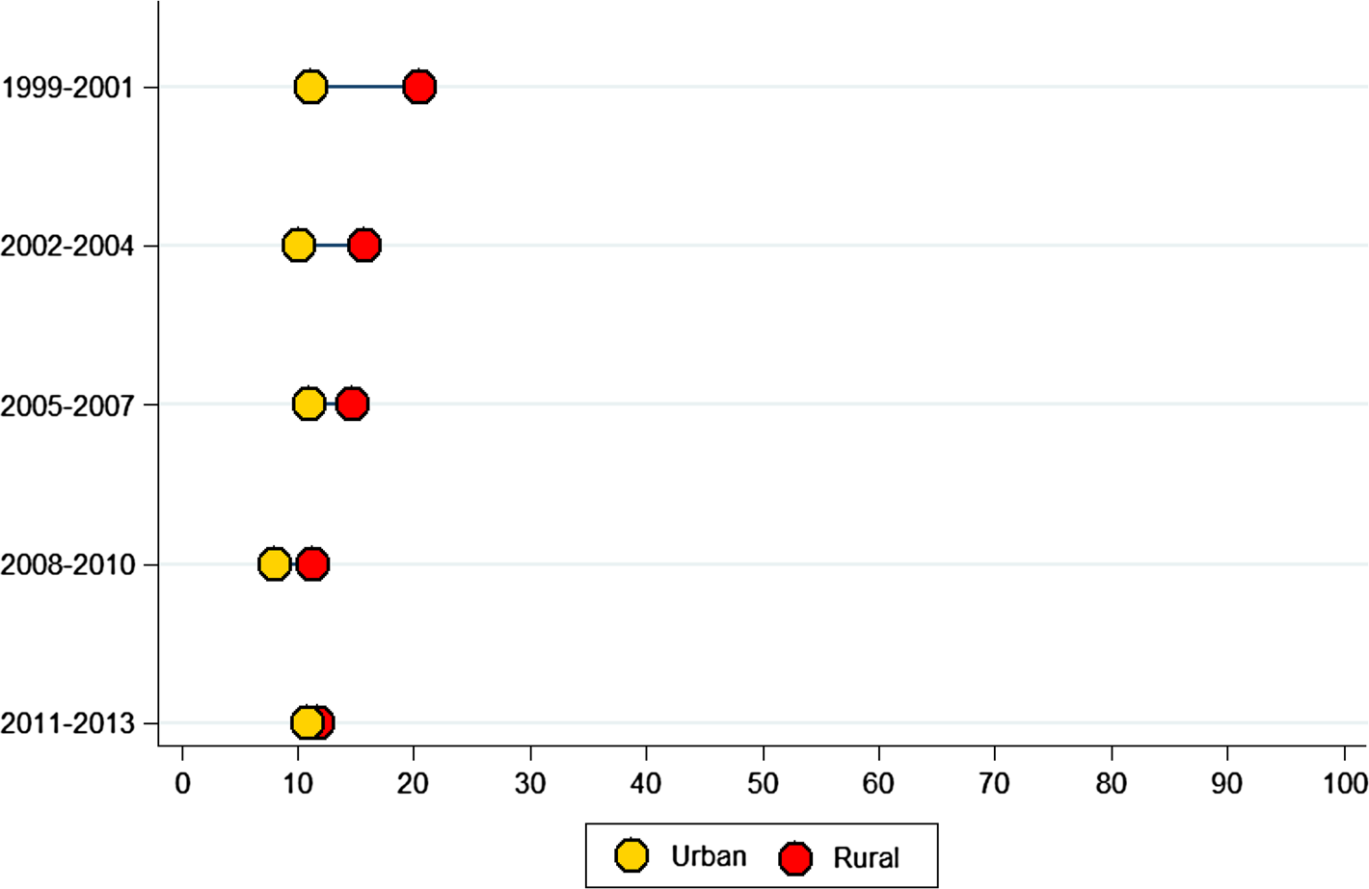
Neonatal mortality rate (Demographic and Health Survey-based birth cohorts) by urban and rural residence in Peru

For the period 1999–2001, the urban and rural areas had NMR of 11.2 and 20.5 per 1,000 live births, respectively. The rates for the period 2011–2013 were 10.8 and 11.8 per 1,000 live births, respectively (Fig. 7).

When compared to the magnitude of improvement over time within each group, differences between baseline and most recent year for coverage increase of NMR-related interventions and for NMR decrease were all significant in the rural area and in the poorest quintile (Table 3). The differences over time were also significant in the urban area and in the richest quintile, except for family planning that was not different within each group, and for NMR that was not different within the richest quintile (Table 3). The poorest quintile and the rural areas performed significantly better than the richest quintile and the urban areas in terms of average annual increase of family planning, antenatal care and skilled birth attendance, and in terms of average annual decrease of NMR, although the difference between wealth quintiles for NMR fell only marginally short of statistical significance (Table 3 and Table 4).

**Table 3:** Neonatal mortality rate (NMR) and coverage of NMR-related interventions in Peru by area of residence and wealth quintile over time

		Urban	95 % CI		*P*-value	Rural	95 % CI		*P*-value	Poorest quintile	95 % CI		*P*-value	Richest quintile	95 % CI		*P*-value
	Coverage in 2000 (%)	90.9	90.1	91.8		81.6	79.9	83.3		75.4	73.0	77.7		93.1	91.6	94.7	
FPS	Annual rate of increase (%)^a^	0.04	−0.06	0.14	NS	0.5	0.3	0.7	<0.001	0.9	0.6	1.2	<0.001	0.1	−0.08	0.3	NS
	Coverage in 2013 (%)	89.7	88.6	90.6		88.2	87.0	89.3		85.5	83.9	87.0		92.2	89.6	94.2	
	Coverage in 2000 (%)	81.1	79.5	82.7		50.9	48.1	53.8		42.9	39.8	45.9		93.3	91.2	95.4	
ANC	Annual rate of increase (%)^a^	0.9	0.8	1.1	<0.001	2.5	2.2	2.9	<0.001	3.9	3.4	4.4	<0.001	0.4	0.2	0.6	<0.001
	Coverage in 2013 (%)	96.6	95.9	97.2		91.5	89.9	92.8		89.1	87.1	90.9		98.3	96.1	99.3	
	Coverage in 2000 (%)	85.0	83.3	86.8		28.7	26.1	31.3		20.1	17.9	22.3		98.4	97.4	99.4	
SBA	Annual rate of increase (%)^a^	0.8	0.7	1.0	<0.001	6.9	6.0	7.8	<0.001	9.6	8.5	10.7	<0.001	−0.05	−0.01	−0.08	0.002
	Coverage in 2013 (%)	97.3	96.4	96.4		71.3	67.1	75.3		65.6	61.1	69.9		99.7	99.0	99.9	
	Rate in 2000 (deaths/1,000)	15.1	12.4	17.9		31.0	27.6	34.4		30.9	26.7	35.2		9.8	4.0	15.5	
NMR	Annual rate of reduction (%)^a^	3.2	1.1	5.2	0.001	6.6	5.2	8.0	<0.001	5.9	4.2	7.6	<0.001	0.9	−6.0	7.3	NS
	Rate in 2013 (deaths/1,000)	11.4	8.3	14.5		13.3	10.4	16.1		14.7	11.1	18.2		7.6	1.3	14.0	

*FPS* family planning needs satisfied, *ANC* at least 4 antenatal care visits, *SBA* Skilled birth attendance

^a^Taking into account all Demographic and Health Surveys from 2000 to 2013

**Table 4:** Comparison of coverage of NMR-related interventions and neonatal mortality rate (NMR) between geographic areas and between wealth quintiles

		Urban vs. rural	Richest quintile vs. Poorest quintile
FPS	Annual rate of increase (%)	<0.001	<0.001
ANC4	Annual rate of increase (%)	<0.001	<0.001
SBA	Annual rate of increase (%)	<0.001	<0.001
NMR	Annual rate of reduction (%)	0.002	0.09

*FPS* family planning needs satisfied, *ANC4* at least 4 antenatal care visits, *SBA* skilled birth attendance

### Completeness of registered neonatal mortality at district-level

**Fig. 8:**
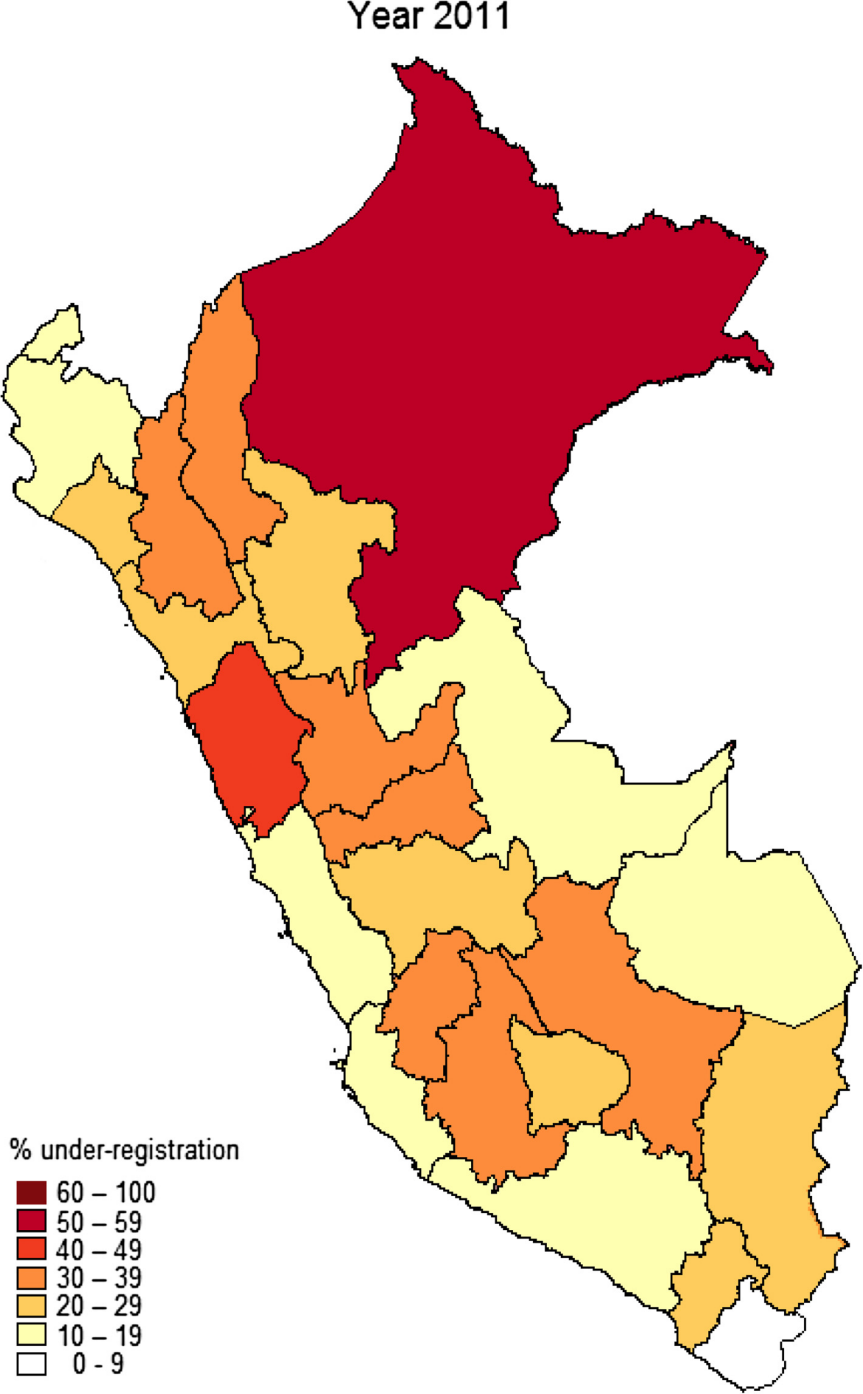
Percentage of under-registered neonatal deaths at departmental level in Peru (2011) [16]

Figure 8 shows the percentage of under-registered neonatal deaths at district-level for year 2011 [17]. Out of 24 districts, only one (Tacna) had less than 9 % of under-registration. Conversely, two (Loreto and Ancash) had more than 40 %, fourteen had between 20–39 %, and seven had between 10–19 % of under-registration. The districts with lower HDI and lower urban population tended to show higher under-registration. The bivariate correlation coefficient was −0.61 (*p* = 0.001) between HDI and under-registration, and −0.6 (*p* = 0.002) between urban population and under-registration.

### Expenditure on maternal and neonatal health interventions

National per capita expenditure on reproductive health in US$ per woman of reproductive age increased from US$ 1.07 in 2002 to US$ 6.35 in 2013, with a transient decline from 2003 to 2008. The pace of increase was higher from 2009 onwards.

Per capita expenditure on maternal and neonatal health in US$ per pregnant woman increased from US$ 34.24 in 2000 to US$ 577.36 in 2013.

### Potential underlying factors

Simple linear regressions showed significant associations between neonatal mortality and each individual predictor of different dimensions of our conceptual framework, including social determinants, access to services, NMR-related health expenditure and coverage of NMR-related interventions, which were all in the expected direction, that is improvement of social determinants and increase in expenditure and in coverage of interventions were inversely associated with the NMR, (Additional file 1: Table S3). However, the coefficients for multiple regressions did not show significant associations, except for urbanisation and skilled birth attendance (Additional file 1: Table S3). The period of analysis considered for the regressions was 2000–2012 and therefore, in the best scenario, we included 13 years, as for some predictors only 10 years were included, given the data availability.

Synthesis of group discussions, individual interviews and the desk review revealed diverse driving factors that may explain the progress achieved by Peru. They are supported by macro-level policy and system changes that occurred over time in Peru, described in detail in Table 5. In summary, a changing political scenario resulted in a definitive transition to democracy in the 2000s, which ran in parallel with the implementation of programmes progressively centred on mother’s and children’s health. These changes were occurring within a context of substantial improvement in social determinants. Newborn health emerged as a prominent area in the political priorities more recently, influenced by civil society organisations e.g., National Agreement, Roundtable Against Poverty, and Colectivo de Salud Neonatal ([24–27], http://www.k4health.org/toolkits/alianzas-neonatales/estudio-de-caso-el-colectivo-de-salud-neonatal-peru), which pushed forward the notion of health as a fundamental right and maternal and neonatal health as a specific and integrated goal that should be considered as an equity issue, by taking into account cultural aspects and a rights-based approach [28].

**Table 5:** Major policy and programmatic changes related to maternal, newborn and child health in Peru (1970-present)

	1970s	1980s	1990s	2000s	2010- to date
Political background	Military dictatorships	Transition to democracy (1980) Social violence (Shining Path: 1982–1992) with rural areas most affected	Continued democratic period, followed by an auto-coup (1992) and political instability Shining Path defeat (1992) Weak public and civil society institutions	Consolidation of democratic system and decentralisation process with mixed results Strengthened participation of civil society in policymaking and accountability	Stable democracy with continued decentralisation process with mixed results Continued strengthened participation of civil society in policymaking and accountability
Economic growth	Economic stagnation	Inflation and foreign debt crisis Economic stagnation and recession Poverty increase	Control of inflation, sustained economic growth and GDP increase Poverty reduction Persistence of social and economic inequities	Fast economic growth and GDP increase. Further poverty reduction. Persistence of social and economic inequities.	Fast economic growth and GDP increase. Further poverty reduction. Efforts to reduce the equity gaps. Social inclusion policies. Rural areas and indigenous populations still lagging behind.
Cross-cutting contextual policies	Agricultural reform Nationalisation of main economic industries	Economic liberalisation Formal restoration of civil rights, but with crisis of governance	Re-insertion into the international financial community Anti-terrorist policies implemented	Consolidation of market economy	Market economy consolidated. Efforts to increase investment and exports and to reach economic diversification. Extension of road infrastructure Public sector reform (including education and health) for improving efficiency and equity
Macro health policies & systems	Establishment of 3-tiered system: Ministry of health and charitable services for poor (“Beneficencias”), social security and private	Continued implementation of 3-tiered system: Ministry of health and charitable services for poor (“Beneficencias”), social security and private	Creation of Maternal-Infant Insurance programme (1998) Continued implementation of 3-tiered system Health system strengthening efforts	Creation of Comprehensive Health Insurance (SIS) (2002) Continued implementation of 3-tiered system Health system strengthening efforts	Universal Health Insurance (AUS) (2009–2010) Continued implementation of 3-tiered system Health system strengthening efforts
Demographic factors	Slow urbanisation Decrease in fertility rate	Urbanisation Decrease in fertility rate	Urbanisation Decrease in fertility rate	Urbanisation Decrease in fertility rate	Urbanisation Decrease in fertility rate
Maternal & neonatal health programmes	None	None	Maternal-Infant Health Insurance programme (1998) Family planning & contraceptive initiatives (government and NGOs) Debate about violation of women’s reproductive and sexual rights.	Expansion of SIS (2002), with focus on under-5 children and mothers Cross-cutting programmes: introduction of Strategic Maternal-Neonatal Programme (2007) Stagnation of sexual & reproductive programmes	Cross-cutting programmes: scaling-up of Strategic Maternal-Neonatal Programme (2010) Stagnation of sexual & reproductive programmes
Child health programmes	Establishment of National Immunisation Programme (1972)	Consolidation of child health vertical programmes and national immunisation campaigns Focus on child survival with low visibility of mothers and newborns	Consolidation of child vertical programmes and national immunisation campaigns IMCI introduction (1996) Focus on child survival with low visibility of mothers and newborns	Expansion of SIS (2002), with focus on children under-5 and mothers Cross-cutting programmes: introduction of Articulate Nutritional Programme (2007)	Expansion to SIS, with focus on under-5 children and mothers Cross-cutting programmes: scaling-up of Articulate Nutritional Programmes (2010)
Non-health sector programmes	Establishment of National Program of Food Support (ONAA) and Programme of Direct Aid (PAD) Limited expansion of water supply & sanitation	Continuity of food supplementation programmes (PRONAA) Expansion of water supply & sanitation, with rural areas lagging behind	Continuity of food supplementation programmes (PRONAA) Expansion of water supply & sanitation, with rural areas lagging behind Improvement of women’s education	Introduction of conditional cash transfer programme (JUNTOS) (2005) Cross-cutting programmes (results-based budgeting) Continuity of food supplementation programmes (PRONAA) Expansion of water supply & sanitation, with rural areas lagging behind Improvement of women’s education	Scaling-up of JUNTOS Integration of social assistance programmes (MIDIS) & further emphasis on social inclusion beyond anti-poverty programmes Improvement of women’s education Non-health sector cross-cutting programmes (results-based budgeting)

Political will was also facilitated by informed technical cadres boosting the use of scientific evidence for reduction of neonatal mortality [29]. Likewise, the Ministry of Finance led the introduction of crosscutting results-based budgeting in several areas including health, in coordination with other sectors including the Ministry of Health, the Ministry of Education, the Ministry of Development and Social Inclusion and the Regional Governments (http://www.mef.gob.pe/contenidos/presu_publ/documentac/generales/PRESUPUESTO_POR_RESULTADOS.pdf). This resulted in the implementation of the Strategic Maternal and Neonatal Health Programme since 2007, which was scaled-up since 2009 ([30], http://www.mef.gob.pe/index.php?option=com_content&view=article&id=2144:salud-materno-neonatal&catid=211&Itemid=101528). This approach has promoted budget allocation based on effective implementation of evidence-based RMNCH interventions in poorest areas, and on achievement of concrete progress in coverage and impact indicators, particularly those related to reduction of maternal and newborn deaths. More recently, emphasis was placed on inclusion of quality indicators for interventions such as antenatal care and skilled birth attendance in the DHS as part of a more effective monitoring [31, 32]. Strengthening capacity of the health system to effectively address complicated deliveries and sick newborns needing more sophisticated care is also being emphasised at national and local levels [30].

## Discussion

Remarkable reductions of NMR have been achieved in Peru, with the greatest reductions in the rural areas and poorest quintiles. Reductions for NMR have been achieved within a framework of sustained economic growth and reduction of poverty, sustained political commitment and leadership, strong civil society advocacy, and of crosscutting integrated implementation of effective RMNCH interventions with a strong component of culturally appropriate antenatal and delivery practices.

In agreement with recent evidence suggesting that NMR reduction is more related to improvement in social determinants than to increase of specific interventions coverage [33], and to health system strengthening-related changes [7], our study suggests that a combination of improvement of contextual factors, non-health sector changes such as scaling-up of an antipoverty conditional cash transfer programme that incentivises health service utilisation [34], and an equitable increase in coverage of interventions may be a powerful and sustained way to reduce NMR. Improvement in social determinants and out-of-health sector factors is necessarily mediated by enabling health policy and system innovation, instrumental for implementation of comprehensive integrated maternal and neonatal interventions, including strengthening of obstetric and neonatal solving capacity of health facilities [7], to cope with complex problems such as pregnancy and delivery complications, and high risk newborns. Peru has achieved substantial progress in such efforts [7, 35], and has also been placing progressively more emphasis to attraction and retention of qualified health professionals, and to putting in place general and neonatal intensive care units across the country, although this is still largely an ongoing effort [36].

During the early 2000s, as a result of civil society demand, poverty roundtables and other institutionalised spaces of dialogue were established, which facilitated muti-sectoral dialogue and citizen participation in the design of social policies, and increased transparency, governance and accountability in the implementation and evaluation of policies and programmes. Within this context of strengthened civil society participation, an increased awareness on the importance of avoiding preventable maternal and neonatal deaths led to implementation of cross-cutting programmes such as the Strategic Maternal-Neonatal Programme, which was introduced in 2007 and scaled-up shortly afterwards (http://www.mef.gob.pe/index.php?option=com_content&view=article&id=2144:salud-materno-neonatal&catid=211&Itemid=101528). The sustained emphasis on a rights-based approach to pregnancy and delivery that took into account respect for cultural diversity has also surely played an important role in the progress achieved, by promoting culturally appropriate practices. Such practices include maternity waiting homes for pregnant women and vertical deliveries with the husband’s or partner’s presence in the delivery room, and are particularly important in rural areas of the Amazon and the Andes of Peru to increase utilisation of health facilities for maternal and neonatal care [28, 37–39]. Financing also played a key role in the progress achieved in neonatal health in Peru, as is attested by an impressive increase of domestic expenditure on reproductive health from 2002 to 2013, and on maternal and neonatal health from 2000 to 2013, by about 9 and 25 times respectively.

Although Peru has decreased the coverage and NMR gap between rural and urban areas and between the richest and the poorest segments of the population, there is still a remaining gap, particularly for facility-dependent interventions such as antenatal care and skilled birth attendance. Similarly, neonates born into wealthier and urban families still show a substantial survival advantage, which should be addressed to achieve further and sustainable reductions in NMR, as a recent study performed in countries of low and middle income suggested [40].

More specifically, in Peru maternal and neonatal health interventions have been implemented as part of an integrated and synergistic approach, confirming an increasing trend within countries committed to push forward an effective convergence to reach real progress in RMNCH [41].

Even if all causes of neonatal deaths were reduced by about 50 % from 2000 to 2013, all infectious and related causes account for a quarter of all neonatal deaths, while pre-term birth complications account for about 44 % and intra-partum related events and congenital abnormalities for a third of the total neonatal mortality. It must be emphasised that deaths occurring within the first week of birth account for about three quarters of all neonatal deaths, highlighting the need to address the main causes of early neonatal mortality. Hence the Peruvian health system is facing the double challenge of successfully tackling traditionally prevalent neonatal causes of death such as infections, as well as causes needing further and substantial investment in appropriate infrastructure, equipment and qualified resources such as prematurity and intra-partum related events, for quality provision of both preventative and curative interventions.

Almost all districts reduced their NMR during the study period. Among the few that increased their rates, Lima, Arequipa and La Libertad had already reached low figures, and therefore we cannot exclude statistical fluctuations rather than real increases, given the low number of neonatal deaths in those districts. Of note, Lima is the capital city of Peru, with the highest urbanisation progress and higher access to tertiary level health facilities and highly specialised health professionals, followed by Arequipa and La Libertad, which are also highly urbanised departments. In such settings, NMR may artificially increase, due to improved mortality registration. By contrast, San Martin and Ayacucho, the other departments where NMR increased, have a substantial rural population, and the increases found are very likely showing a remaining urban-rural equity gap that needs to be urgently addressed.

The limitations of this study are that the regressions are exploratory, and therefore cannot prove causal associations to explain the factors behind the progress achieved by Peru. This is due to two main reasons, firstly neonatal mortality reduction is multi-causal where each potential factor is interrelated, and therefore quantitative approaches may not be able to fully explain this complex causality network. Secondly, the number of years for which we had data available is limited, and may account at least partially for the lack of consistent associations in the multiple regression analysis.

Complete and reliable registered data are part of the health system changes needed to effectively monitor the progress of NMR. Peru has recently achieved a substantial reduction in the under-registration of neonatal deaths, due to the implementation of a national surveillance system tracing neonatal deaths at district-level, as well as complete and correct registration [17] - although further improvement is needed, particularly in the less developed departments, which also have higher proportions of rural population. Thus we acknowledge that relying on DHS data for analysing trends and specific patterns of NMR has well known limitations [42]. We tried to overcome this drawback by assembling birth cohorts through pooling of several DHS. We foresee that future analyses using better quality of registered data will soon be possible in Peru, thanks to the described improvements in the vital registration system.

The example of Peru offers useful lessons to low and middle-income countries committed to accomplish the MDGs and post-2015 goals, specifically those related to improvement of maternal and neonatal health, which will need further and substantial reductions of NMR and also securing a healthy start for every newborn to guarantee a future free of disabilities [6].

## Conclusions

In conclusion, Peru has achieved a substantial and equitable reduction of NMR due to a unique combination of improvement of social determinants, political will and commitment, and civil society advocacy, which translated into equitable and sustained implementation of effective maternal and neonatal health interventions. Additional efforts will be needed to close the remaining gaps posed by a double burden of neonatal deaths that encompass traditionally prevalent and more complex causes, which will need a combined implementation of primary care level and high technology interventions such as neonatal intensive care units at national and sub-national levels.

## Abbreviations

CHERG, Child health epidemiology reference group; DHS, Demographic and health surveys; HDI, Human development index; IGME, Inter-agency group for child mortality estimation; IHME, Institute of health metrics and evaluation; LMIC, Low and middle-income countries; MDG, Millennium development goal; NMR, Neonatal mortality rate

## Additional file

Additional file 1: Supplementary tables. (DOCX 80 kb) Additional file 2: Dataset used for the main analyses of this paper. (DTA 259 kb)
