# Supplementary material for: Examining national and district-level trends in neonatal health in Peru through an equity lens: a success story driven by political will and societal advocacy
Source: BMC Public Health. 2016 Sep 12;16(Suppl 2):796. doi: 10.1186/s12889-016-3405-2 (PMC5025833; doi:10.1186/s12889-016-3405-2)
Supplement: Additional file 1: — Supplementary tables. (DOCX 80 kb) [file 12889_2016_3405_MOESM1_ESM.docx]

**Examining national and district-level trends in neonatal health in Peru through an equity lens: a success story driven by political will and societal advocacy**

**Additional file 1**

Table of Contents

A. Table S1. Neonatal mortality rates in Peru by cause (2000-2013) 2

B. Table S2. Early and late neonatal mortality rates (NMR) at departmental level in Peru (2011-2012) 3

C. Table S3. Simple and multilevel linear regressions between Neonatal Mortality Rate (NMR) in Peru and explanatory variables 4

# Table S1. Neonatal mortality rates in Peru by cause (2000-2013)

|  | | | | | | | | |
| --- | --- | --- | --- | --- | --- | --- | --- | --- |
| **Year** | **All causes** | **Pneumonia** | **Preterm birth complications** | **Intrapartum-related events** | **Sepsis/Meningitis/Tetanus** | **Other conditions** | **Congenital abnormalities** | **Diarrhoea** |
| **2000** | 16.2 | 0.9 | 7.0 | 2.8 | 1.9 | 1.2 | 2.4 | 0 |
| **2001** | 15.3 | 0.9 | 6.7 | 2.7 | 1.7 | 1.1 | 2.2 | 0 |
| **2002** | 14.5 | 0.8 | 6.0 | 2.4 | 2.0 | 1.0 | 2.2 | 0 |
| **2003** | 13.7 | 0.8 | 5.6 | 2.3 | 1.8 | 1.0 | 2.2 | 0 |
| **2004** | 12.9 | 0.8 | 5.4 | 2.1 | 1.5 | 1.0 | 2.1 | 0 |
| **2005** | 12.2 | 0.7 | 5.1 | 2.0 | 1.7 | 0.9 | 1.8 | 0 |
| **2006** | 11.5 | 0.7 | 4.6 | 1.8 | 1.4 | 0.9 | 2.1 | 0 |
| **2007** | 10.9 | 0.6 | 4.3 | 1.6 | 1.3 | 0.9 | 2.2 | 0 |
| **2008** | 10.3 | 0.6 | 4.0 | 1.5 | 1.2 | 0.8 | 2.1 | 0 |
| **2009** | 9.7 | 0.5 | 3.8 | 1.4 | 1.1 | 0.8 | 2.1 | 0 |
| **2010** | 9.2 | 0.5 | 3.6 | 1.4 | 1.0 | 0.8 | 2.0 | 0 |
| **2011** | 8.7 | 0.4 | 3.4 | 1.3 | 0.9 | 0.8 | 1.9 | 0 |
| **2012** | 8.3 | 0.4 | 3.2 | 1.2 | 0.9 | 0.7 | 1.9 | 0 |
| **2013** | 8.0 | 0.4 | 3.2 | 1.2 | 0.8 | 0.7 | 1.8 | 0 |

Sources: IGME (UN Inter-agency Group for Child Mortality Estimation); and CHERG (Child Health Epidemiology Reference Group)

# Table S2. Early and late neonatal mortality rates (NMR) at departmental level in Peru (2011-2012)

|  | | | | | |
| --- | --- | --- | --- | --- | --- |
|  | **Total NMR** | **Early NMR (0-7 days)** | | **Late NMR (8-28 days)** | |
|  |  | **Rate** | **%** | **Rate** | **%** |
| **PERU** | 12.8 | 9.3 | 72.7 | 3.5 | 27.3 |
| Amazonas | 14.8 | 12.5 | 84.5 | 2.3 | 15.5 |
| Ancash | 9.7 | 6.3 | 64.9 | 3.4 | 35.1 |
| Apurímac | 10.3 | 8 | 77.7 | 2.3 | 22.3 |
| Arequipa | 7.8 | 5.4 | 69.2 | 2.4 | 30.8 |
| Ayacucho | 14.9 | 8 | 53.7 | 6.9 | 46.3 |
| Cajamarca | 11.3 | 7 | 61.9 | 4.3 | 38.1 |
| Callao | 9.6 | 7.9 | 82.3 | 1.7 | 17.7 |
| Cusco | 20 | 11.4 | 57.0 | 8.6 | 43.0 |
| Huancavelica | 12.1 | 7.0 | 57.9 | 5.1 | 42.1 |
| Huánuco | 18.9 | 14.4 | 76.2 | 4.5 | 23.8 |
| Ica | 11.2 | 9.5 | 84.8 | 1.8 | 16.1 |
| Junín | 15.7 | 10.4 | 66.2 | 5.3 | 33.8 |
| La libertad | 11.5 | 8.7 | 75.7 | 2.8 | 24.3 |
| Lambayeque | 15.3 | 12.1 | 79.1 | 3.2 | 20.9 |
| Lima | 8.2 | 5.9 | 72.0 | 2.3 | 28.0 |
| Loreto | 14.4 | 12.8 | 88.9 | 1.6 | 11.1 |
| Madre de dios | 20.5 | 19.5 | 95.1 | 1.0 | 4.9 |
| Moquegua | 18.8 | 15.5 | 82.4 | 3.3 | 17.6 |
| Pasco | 20.3 | 16 | 78.8 | 4.3 | 21.2 |
| Piura | 15.2 | 11.4 | 75.0 | 3.8 | 25.0 |
| Puno | 15.8 | 11.2 | 70.9 | 4.6 | 29.1 |
| San Martin | 19 | 14.7 | 77.4 | 4.3 | 22.6 |
| Tacna | 13.2 | 10.9 | 82.6 | 2.3 | 17.4 |
| Tumbes | 21 | 18.5 | 88.1 | 2.5 | 11.9 |
| Ucayali | 25.8 | 20 | 77.5 | 5.8 | 22.5 |

Source: Ministry of Health (Ministerio de Salud. Dirección General de Epidemiología (2013). "Mortalidad Neonatal en el Perú y sus Departamentos, 2011 – 2012." Retrieved January 20, 2016, from <http://www.dge.gob.pe/portal/docs/Mortalidad_neonatal11_12.pdf.>)

# C. Table S3. Simple and multilevel linear regressions between Neonatal Mortality Rate (NMR) in Peru and explanatory variables

|  | | | | | |
| --- | --- | --- | --- | --- | --- |
| **Dimension** | **Variables** | **Crude regression coefficient (95% CI)** | **p** | **Adjusted regression coefficient (95% CI)** | **P** |
| **Socioeconomic factors** | GDP per capita (constant 2012 US$) | -0.002 (-0.002 to -0.001) | < 0.001 | 0.000 (0.000 to 0.001) | 0.375 |
|  | Gini for income (%) | 0.562 (0.356 to 0.768) | < 0.001 | -0.083 (-0.206 to 0.041) | 0.145 |
|  | Poverty line (%) | 0.188 (0.098 to 0.278) | 0.001 | -0.008 (-0.027 to 0.010) | 0.292 |
|  | Unmet basic needs (%) | 0.387 (0.334 to 0.441) | < 0.001 | -0.017 (-0.120 to 0.086) | 0.687 |
|  | Urbanisation (%) | -1.123 (-1.186 to -1.059) | < 0.001 | -1.424 (-1.691 to -1.156) | < 0.001 |
| **Out-of-health sector changes** | Median years of schooling, women | -2.456 (-4.494 to -0.418) | 0.024 | -0.723 (-2.796 to 1.350) | 0.388 |
|  | Total fertility rate | 9.344 (-0.868 to 19.556) | 0.068 | 6.338 (-0.580 to 13.256) | 0.064 |
|  | Improved water access (%) | -0.769 (-1.350 to -0.189) | 0.017 | 0.335 (-0.409 to 1.079) | 0.279 |
|  | Cash Transfer Programme coverage (Juntos) (%) | -0.156 (-0.201 to -0.111) | < 0.001 | -0.140 (-0.293 to 0.014) | 0.065 |
| **Health-sector changes** | Health insurance system, SIS (Attendances/under-5 child) | -3.137 (-4.804 to -1.469) | 0.002 | -2.174 (-13.788 to 9.440) | 0.253 |
|  | Density of human resources (per 10,000 population) | -0.623 (-0.888 to -0.358) | 0.003 | 0.184 (-5.224 to 5.592) | 0.741 |
|  | Per capita expenditure on reproductive health, previous year (constant 2012 US$/fertile woman) | -0.708 (-1.264 to -0.151) | 0.018 | -0.536 (-8.667 to 7.594) | 0.556 |
|  | Per capita expenditure on maternal-neonatal, previous year health (constant 2012 US$/pregnant woman) | -0.018 (-0.024 to -0.012) | < 0.001 | 0.001 (-0.096 to 0.098) | 0.93 |
| **Coverage of NMR-related interventions** | Family planning needs satisfied (%) | -1.362 (-2.142 to -0.583) | 0.004 | -0.195 (-0.530 to 0.139) | 0.203 |
|  | Antenatal care visits (%) | -0.292 (-0.373 to -0.211) | < 0.001 | -0.057 (-0.159 to 0.046) | 0.225 |
|  | Skilled birth attendance (%) | -0.270 (-0.309 to -0.232) | < 0.001 | -0.199 (-0.282 to -0.117) | 0.001 |
